# A New 2-(2-Phenylethyl)Chromone from Chinese Eaglewood

**DOI:** 10.3390/molecules14125165

**Published:** 2009-12-09

**Authors:** Hao-Fu Dai, Jun Liu, Yan-Bo Zeng, Zhuang Han, Hui Wang, Wen-Li Mei

**Affiliations:** Key Laboratory of Tropical Crop Biotechnology, Ministry of Agriculture, Institute of Tropical Bioscience and Biotechnology, Chinese Academy of Tropical Agricultural Sciences, Haikou 571101, China; E-Mails: hfdai2001@yahoo.com.cn (H-F.D.); jollyliu1983@163.com (J.L.); zengyanbo@163.com (Y-B.Z.); hanzone@yahoo.cn (Z.H.); wanghuilily2000@163.com (H.W.)

**Keywords:** Chinese eaglewood, *Aquilariasinensis*, chromone

## Abstract

A new 2-(2-phenylethyl)chromone, 5,6,7,8-tetrahydroxy-2-(3-hydroxy-4-methoxyphenethyl)-5,6,7,8-tetrahydro-4*H*-chromen-4-one (**1**) was isolated from the Chinese eaglewood [*Aquilaria* *sinensis* (Lour.) Gilg]. Its structure was established by detailed MS and NMR spectroscopic analysis, as well as comparison with literature data.

## 1. Introduction

Chinese eaglewood is the resin-deposited part of the trunk of *Aquilaria sinensis* (Lour.) Gilg, which has been used in China as incense as well as a traditional sedative, analgesic and digestive medicine [1]. Characteristic sesquiterpenes and chromone derivatives have been isolated from Chinese eaglewood in recent decades [2,3,4]. In our previous screening for cytotoxic agents from tropical medicinal plants, a new cytotoxic 2-(2-phenylethyl)chromone has been isolated from Chinese eaglewood collected in Hainan Province of China [5]. Continued study on the chemical constituents from Chinese eaglewood led to the isolation of a new 2-(2-phenylethyl)chromone, 5,6,7,8-tetra-hydroxy-2-(3-hydroxy-4-methoxyphenethyl)-5,6,7,8-tetrahydro-4*H*-chromen-4-one (**1**). The present paper describes the isolation and structural elucidation of the new compound.

## 2. Results and Discussion

Compound **1** was isolated as pale yellow powder, mp 109–111 °C, [α]_18_^D^ + 12.3 (*c* 1.0, MeOH). The [M+Na]^+^ at *m/z* 387.1053 (calcd. 387.1056) in the high-resolution ESI-Mass spectrum corresponded to the molecular formula C_1__8_H_2__0_O_8_. This formula can also be validated through ^1^H-NMR, ^13^C-NMR and DEPT spectra. Its IR spectrum showed the presence of hydroxyl (3,409 cm^-1^), unsaturated carbonyl (1,652 cm^-1^) and phenyl (1,570, 1,514, 1,456 cm^-1^) groups. The ^1^H-NMR spectrum (Table 1) of **1** showed the presence of one methoxyl group at *δ*_H_ 3.71 (3H, s), four consecutive methine protons [*δ*_H_ 4.48 (1H, d, *J* = 4.2 Hz, H-5), 3.74 (1H, dd, *J* = 6.9, 4.2 Hz, H-6), 3.83 (1H, t, *J* = 6.9, H-7), and 4.31 (1H, d, *J* = 6.9 Hz, H-8)], two methylene groups at *δ*_H_ 2.80 (4H, overlapped, H-7', 8') and one 1,3,4-trisubstituted phenyl group at *δ*_H_ 6.80 (1H, d, *J* = 8.0 Hz, H-5'), *δ*_H_ 6.67 (1H, d, *J* = 1.9 Hz, H-2') and *δ*_H_ 6.60 (1H, dd, *J* = 8.0, 1.9 Hz, H-6'). The ^13^C-NMR spectrum (Table 1) of **1** showed the presence of two methylene groups at *δ*_C_ 31.1 and 34.3, one methoxyl at *δ*_C_ 55.6 and four consecutive methine carbons (*δ*_C_ 64.7, *δ*_C_ 72.7, *δ*_C_ 70.6 and *δ*_C_ 68.4). Based on the above evidence, compound **1** was presumed to be 2-(2-phenylethyl)chromone derivative. The ^13^C-NMR spectra of **1** was similar to that of the 8-chloro-5,6,7-trihydroxy-2-(3-hydroxy-4-methoxyphenethyl)-5,6,7,8-tetrahydro-4*H*-chromen-4-one [5] except that C-8 was substituted by a hydroxyl group instead of a chlorine atom. The relative stereochemistry was determined by ^1^H-^1^H coupling constants. The relatively small coupling constant between H-5 and H-6 revealed the *cis* relationship between H-5 and H-6 [6]. While the relatively large coupling constants between H-6 and H-7, H-7 and H-8, revealed the *trans* relationships between them [7]. The relative stereochemistry of compound **1** was also confirmed by the ROESY experiment. In the ROESY spectrum, the cross peaks from *δ* 4.31 (H-8) to *δ* 4.48 (H-5) and 3.74 (H-6) indicated that H-5, H-6 and H-8 were at the same side. While the cross peak from *δ* 4.48 (H-5) to *δ* 3.83 (H-7) was not observed, which indicated H-5 and H-7 were at the different side. Consequently, the structure of **1** was established as 5,6,7,8-tetrahydroxy-2-(3-hydroxy-4-methoxyphenethyl)-5,6,7,8-tetrahydro-4*H*-chro- men-4-one. The result of bioactive assay showed that compound **1 **exhibited no cytotoxic activity against K562, SGC-7901, SMMC-7721 cell lines. 

**Figure 1:**
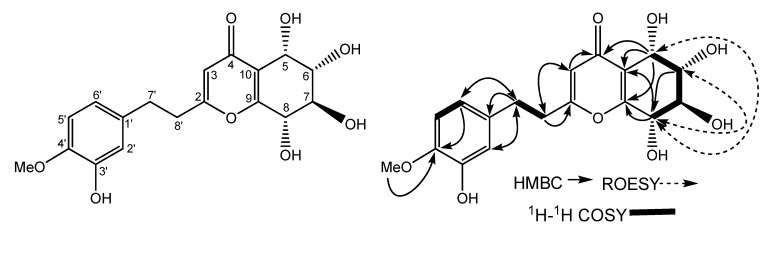
Structure and key correlations of compound **1**.

**Table 1:** NMR data of **1** in DMSO-*d*_6_ (^1^H: 400MHz; ^13^C: 100 MHz; *δ* in ppm, *J* in Hz).

Position	δ_H_	δ_C_	Position	δ_H_	δ_C_
2		168.0	1'		132.6
3	6.07 (1H, s)	112.7	2'	6.67 (1H, d, 1.9)	115.6
4		178.5	3'		146.0
5	4.48 (1H, d, 4.2)	64.7	4'		146.3
6	3.74 (1H, dd, 6.9, 4.2 )	72.7	5'	6.80 (1H, d, 8.0)	112.2
7	3.83 (1H, t, 6.9)	70.6	6'	6.60 (1H, dd, 8.0, 1.9)	118.7
8	4.31 (1H, d, 6.9)	68.4	7'	2.80 (2H, overlapped)	31.1
9		163.1	8'	2.80 (2H, overlapped)	34.3
10		120.7	4'-OCH_3_	3.71 (3H, s)	55.6

## 3. Experimental

### 3.1. General

Melting points were obtained on a Beijing Taike X-5 stage apparatus and are uncorrected. Optical rotation was recorded using a Rudolph Autopol III polarimeter (Rodolph Research Analytical, New Jersey, USA). The IR spectra were obtained on a Nicolet 380 FT-IR instrument, as KBr pellets. The NMR spectra were recorded on a Bruker AV-400 spectrometer, using TMS as an internal standard. The HRESIMS spectra were measured with an API QSTAR Pulsar mass spectrometer. Column chromatography was performed with silica gel (Marine Chemical Industry Factory, Qingdao, P. R. China), Sephadex LH-20 and Rp-18 gel (Merck, Darmstadt Germany). TLC was preformed with silica gel GF254 (Marine Chemical Industry Factory, Qingdao, P. R. China).

### 3.2. Plant Material

The material of Chinese eaglewood was collected in Ding’an county of Hainan Province, China, in May 2006, and the material was identified by Professor Hao-Fu Dai. A voucher specimen (No. CX20060501) is deposited in the Institute of Tropical Bioscience and Biotechnology, Chinese Academy of Tropical Agricultural Sciences.

### 3.3. Extraction and Isolation

The material of Chinese eaglewood (35.4 Kg) was exhaustively extracted with 95% EtOH three times at room temperature and filtered. After evaporation, the residue was suspended in H_2_O and partitioned with EtOAc to afford EtOAc extract. The H_2_O part was applied to a D101 reticular resin column eluted with H_2_O and MeOH. The MeOH eluent was concentrated *in vacuo* to give a residue (147.3 g), which was chromatographed on a silica gel column (200-300 mesh) with gradient elution utilizing CHCl_3_-MeOH as solvent system to give nine fractions. Fraction 7 (25.8 g) was chromatographed on a RP-18 column with gradient elution of MeOH-H_2_O as solvent system to give 10 fractions (Fr.7-1~10). Fraction 7-7 (4.1 g) was subjected to column chromatography over Sephadex LH-20 eluted with 95 % EtOH and further purified by silica gel column chromatography eluted with CHCl_3_-MeOH (9:1) to afford *5,6,7,8-tetrahydroxy-2-(3-hydroxy-4-methoxyphenethyl)-5,6,7,8-tetra-hydro-4H-chromen-4-one* (**1, **119 mg). Pale yellow powder, C_18_H_20_O_8_, mp 109–111 °C, [α]_18_^D^ + 12.3 (*c* 1.0, MeOH). IR (KBr) ν_max_ (cm^−^^1^): 3,409, 1,652, 1,570, 1,514, 1,456, 1,285, 1,110, 1,049. ^1^H- and ^13^C-NMR spectral data: see Table 1; HREI-ESI-MS *m/z*: 387.1053 [M+Na]^+^ (calcd. for C_1__8_H_2__0_O_8_Na, 387.1056). 

## 4. Conclusions

As a part of our chemical investigation on Chinese eaglewood [*Aquilaria* *sinensis* (Lour.) Gilg], a new 2-(2-phenylethyl)chromone, 5,6,7,8-tetra-hydroxy-2-(3-hydroxy-4-methoxyphenethyl)-5,6,7,8-tetrahydro-4*H*-chromen-4-one (**1**) was isolated. Its structure was established on the basis of spectroscopic evidence. The result of bioactive assay showed that compound **1 **exhibited no cytotoxic activity against K562, SGC-7901, SMMC-7721 cell lines.
